# Efficient GSH delivery using PAMAM-GSH into MPP-induced PC12 cellular model for Parkinson’s disease

**DOI:** 10.1093/rb/rbw032

**Published:** 2016-09-22

**Authors:** Hong-Ji Sun, Yan Wang, Tong Hao, Chang-Yong Wang, Qi-Yu Wang, Xiao-Xia Jiang

**Affiliations:** Department of Advanced Interdisciplinary Studies, Institute of Basic Medical Sciences and Tissue Engineering Research Center, Academy of Military Medical Sciences, 27 Taiping Road, Haidian District, Beijing 100850, People’s Republic of China

**Keywords:** polyamidoamine, glutathione, MPP, PC12 cells, Parkinson’s disease

## Abstract

Glutathione (GSH) depletion has been an important contributor to the dysfunction of dopamine neurons. Polyamidoamine-GSH (PAMAM-GSH) was synthesized and the delivery effect of GSH into PC12 cells was tested. MTT assessment for cytotoxicity and reactive oxygen species (ROS) as well as nitrite oxide (NO) and intracelluar superoxide dismutase (SOD) detection for antioxidative ability were performed. Furthermore, the antiapoptotic ability was analysed by assessing caspase-3, JNK1/2 and Erk1/2 expression. Our data indicated that PAMAM-GSH is an effective agent to replenish GSH into PC12 cells. PAMAM-GSH developed its antioxidative and protective ability for 1-methyl-4-phenylpyridinium (MPP)-induced PC12 cells by reducing the intracellular levels of ROS and SOD activity as well as decreasing the release of NO. Meanwhile, PAMAM-GSH could inhibit caspase-3 activation and might show its antiapoptotic ability to MPP-induced PC12 cells through JNK2/Erk1/2 pathway. In summary, these studies suggest that PAMAM-GSH conjugate has an intrinsic ability to penetrate PC12 cells and deliver GSH into these cells which may provide a new strategy for clinical applications in the treatment of Parkinson’s disease.

## Introduction

Parkinson’s disease (PD) is a common progressive neurodegenerative disease, which is characterized by clinical features including bradykinesia, rigidity, tremor and postural instability [1] as well as loss of dopamine-secreting neurons within substantia nigra [2] and the accumulation of misfolded α-synuclein into intracellular aggregates called Lewy bodies [3, 4]. Parkinson’s disease. Though the etiology of PD is still not completely clear, several causes have been found responsible, which include genetic mutations of several genes [5–7], oxidative stress [8], mitochondrial dysfunction [9], lysosomal and proteasomal dysfunction [10, 11] as well as glutathione (GSH) depletion [12]. And among these causes, GSH depletion has been an important contributor to the dysfunction of dopamine neurons, which results in more vulnerable for oxidative injury and disturbing the neuromodulator role of GSH in dopamine neurons [13].

GSH plays an important role as antioxidant and neuromodulator in the central nervous system. And GSH itself or combined with other enzymes can perform cleavage ability of intracellular free radicals, such as superoxide radicals, hydroxyl radicals and peroxynitrites [14]. At the same time, GSH protects against glutamate excitotoxicity as a neuromodulator. As is synthesized in neurons and astrocytes, GSH plays its antioxidative and antiapoptotic ability mostly inside the cells [15]. Since GSH depletion is one of the main causes of PD and contributes to the dysfunction of dopamine neurons, some researchers have focused the therapy for PD on how to replace the function of GSH by other antioxidants or how to restore the GSH concentration inside dopamine neurons. However, the effects of previous research are not obvious. Although antioxidants used in some studies have shown some protective effects against PD, they could not replace the neuromodulator role of GSH inside dopamine cells [16–19]. And other researchers have developed several ways to deliver GSH into the central nervous system which includes analogues [20], prodrugs and codrugs [21, 22] as well as liposomes [23] and nanoparticles [24, 25]. However, the strategies used before have either lacked the ability to cross the cellular membrane of neurons or showed low efficiency for GSH intracellular delivery. So how to restore GSH concentration inside dopamine neurons to the physiological level with high efficiency has been the main issue for GSH therapy for PD.

Because of its well-defined architecture, multivalency and versatility [26], Polyamidoamine (PAMAM) dendrimer has been an effective vehicle for drug delivery [27], gene therapy [28] and contrast agent [29]. And it has been used in the research of treatments to several central nervous systems diseases, such as ischemia/reperfusion injury [30], PD [31, 32], malignant glioma [33], Alzheimer’s disease [34].

In order to restore the GSH function as an antioxidant and neuromodulator inside dopamine neurons of PD, we took advantage of the transmembrane ability and high load efficiency of PAMAM dendrimer to deliver and replenish GSH. In the present study, PAMAM-GSH conjugates were synthesized. The successful combination of GSH to PAMAM was characterized. Meanwhile, PAMAM-GSH was evaluated for its delivery efficiency of GSH and its cytotoxicity, antioxidative ability as well as antiapoptotic ability in the cellular model of PD.

## Materials and methods

### Materials

Generation 4.0 PAMAM dendrimer was obtained from Weihai CY Dendrimer Technology Co., Ltd. And N-hydroxysuccinimide-PEG_2_-maleimide (SM(PEG)_2_) was purchased from Jenkem Technology Co., Ltd. Fluorescein was from Sigma and Glutathione was from Aladdin Chemistry Co., Ltd.

### Cell culture

PC12 cells were cultured in DMEM medium (Gibco) supplemented with 5% horse serum, 10% fetal bovine serum, 100U/mL penicillin and 100 μg/mL streptomycin in a humidified atmosphere of 5% CO_2_ at 37°C.

### Preparation and characterization of G4.0 PAMAM-GSH conjugates

PAMAM-GSH conjugates were prepared in a six-step sequence. First, PAMAM-SM(PEG)_2_ was synthesized through the reaction of G4.0 PAMAM dendrimer and SM(PEG)_2_ in phosphate buffered saline (PBS, pH 7.4) for 2 h at room temperature. The compound obtained from the first step was filtered by ultrafiltration tube (MWCO 10000, Millipore, Bedford, MA Millipore) using ultra centrifuge (Sigma 3K18) to get rid of unreacted SM(PEG)_2_ and deionized water. Then the product of PAMAM-SM(PEG)_2_ was resolved in PBS (pH 7.0) and reacted with GSH for 24 h in room temperature. Especially, additional 1% fluorescein was added to the reactive system in order to obtain FITC-Labeled PAMAM-GSH. The final conjugate of PAMAM-GSH was obtained through dialysis by cellulose membranes (MWCO 3000, Union Carbide, NY) for 48 h and lyophilization for 72 h. According to calculation, the substituent degree of GSH in PAMAM-GSH conjugates was 15% theoretically. And the characterization of PAMAM-GSH was conducted by ^1^H NMR (INOVA, Varian, USA), Fourier transform infrared (FTIR) spectroscopy (NICOLETNexus-470 ARK FTIRUSA).

### Cytotoxicity of PAMAM-GSH

Cellular cytotoxicity was assessed by 3-(4,5-dimethylthiazol-2-yl)-2,5-diphenyl-tetrazolium bromide (MTT) assay. PC12 cells were seeded into 96-well plate at 5 × 10^3^ cells/well, and incubated at 37°C. When it came to 70% confluence, different concentrations of PAMAM dendrimer and PAMAM-GSH (0.25, 1, 4 μM) were added into the culture medium for a further 24 h before MTT assay. For MTT assessment, 10 μM MTT was added into the culture medium and incubated for another 4 h before measuring at 490 nm using a spectrophotometer (Molecular Device).

### Flow cytometry analysis

PC12 cells were seeded into a 60 mm culture plate at the 1 × 10^6^ cells/mL seeding density using the normal culture medium. When it came to 70% confluence, PC12 cells were treated with 1 μM FITC-labeled PAMAM-GSH for 1, 2, 3, 4, 5, 6, 12 and 24 h before being collected for flow cytometry analysis (FCA). The cells were washed with PBS (pH 7.4) for three times, trypsinized and centrifuged at 1000 rpm for 5 min to obtain a cell pellet. And then the cells were resuspended with 2% para-formaldehyde and analyzed using a flow cytometer (FACSCaliber, BD) by counting 20 000 events. The mean fluorescence intensity of cells was calculated using the software (Cell Quest Pro).

### Confocal microscopy

PC12 cells were seeded onto glass (10 × 10 mm) at 4 × 10^4^ cells/mL before it came to 70% confluence. And the glass was pre-treated with 0.05% Poly-l-Lysine (Sigma) for 2 h. Then PC12 cells were treated with 1 μM FITC-labeled PAMAM-GSH for 6 and 12 h before being washed with PBS for three times and fixed with 2% para-formaldehyde for 30 min. PC12 cells were then treated 0.05 mg/mL propidium iodide (PI) for nuclear staining and washed with PBS for three times before capturing using a confocal microscope (Zeiss LSM 310). The wavelengths used were 488 nm for FITC-fluorescein and 543 nm for PI, respectively.

### Transmission electron microscopy

PC12 cells were seeded into 60 mm culture plate at 1 × 10^6^ cells/mL before it came to 70% confluence. And then they were treated with 1 μM PAMAM-GSH for 6 and 12 h before sample preparation of Transmission Electron Microscopy (TEM, Philip Technai 10). The pictures were taken by the AMT camera system in the condition of HV-80.0KV.

### Intracellular GSH detection

PC12 cells were seeded into 60 mm culture plate at 1 × 10^6^ cells/mL before it came to 70% confluence. Then they were treated with different concentrations of PAMAM-GSH and PAMAM dendrimer (0.25, 1, 4 μM) for 24 h. The cells were collected after washing with PBS for three times and treated with frozen-melt method for several times before intracellular GSH assessment using 5,5′-Dithiobis-(2-nitrobenzoic acid) (DTNB) method [35].

### Preparation of the cellular model of PD

PC12 cells were seeded into 96-well plate at 5 × 10^3^ cells/mL overnight. And then they were treated with different concentrations of 1-methyl-4-phenylpyridinium (MPP) to induce oxidative stress injury for 24 and 48 h. The concentrations of MPP used in the explorative experiment were 0, 125, 250, 500, 1000 and 2000 μM. The activity of metabolic viability of PC12 cells was assessed using 3-(4,5-dimethylthiazol-2-yl)-2,5-diphenyl tetrazolium bromide (MTT) method as described before and their cellular viability was detected with Acridine orange/Propidium iodide (AO/PI) staining which performed according to the data sheet.

### Antioxidative assessments of PAMAM-GSH to PC12 cells

Antioxidative was assessed by the detection of reactive oxygen species (ROS), the release of nitrite oxide (NO) and the activity of intracelluar superoxide dismutase (SOD) according to the instructions (Beyotime, China).

Simply, PC12 cells were seeded into 96-well plate overnight at 5 × 10^3^ cells/mL for ROS and NO assessment while 60 mm culture plate at 1 × 10^6^ cells/mL for SOD assessment. And then they were treated with different concentrations of PAMAM-GSH for 24 h before another 48 h treatment of 500 μM MPP. The concentration of PAMAM-GSH used here were 0.25, 1 and 4 μM.

For intracellular ROS assessment, 10 μM 2′,7′-dichlorofluorescin diacetate (DCFH-DA) (Beyotime, China) method was used for intracellular ROS detection according to the instruction. After incubated with 10 μM DCFH-DA for 20 min, PC12 cells were washed with PBS for three times. And the pictures were obtained by fluorescence microscope (Olympus). The fluorescence intensity of DCFH-DA staining was evaluated by fluorescence spectrophotometer (Molecular Device). The excitation and emission wavelengths used were 488 and 525 nm, respectively.

For NO detection, the supernatant was collected and transferred to another 96-well plate. Then NO released from PC12 cells was measured at 540 nm using spectrophotometer according to the manufacturer’s instructions (Beyondtime).

For SOD assessment, proteins were extracted and quantified before activity of SOD measurement at 450 nm using the commercial kit (Beyondtime).

### Antiapoptotic assessment of PAMAM-GSH to PC12 cells

PC12 cells were seeded into 60 mm culture plate at 1 × 10^6^ cells/mL overnight and then pre-treated with different concentrations of tPAMAM-GSH for 24 h before another 48 h treatment with 500 μM MPP. PC12 cells were collected before protein extraction for western blot analysis of caspase-3, cleaved caspase-3, JNK1/2 and Erk1/2.

### Western blotting

Extracted proteins were loaded on 12% SDS polyacrylamide gel and separated by electrophoresis for 1 h and then were transferred to a PVDF membrane (Roche). And the membrane was blocked with 5% defatted milk for 1 h at room temperature and then incubated with primary antibody overnight at 4°C (rabbit anti-caspase-3, 1:1000; rabbit anti-cleaved caspase-3, 1:1000; rabbit anti-JNK1/2, 1:1500; rabbit anti-Erk1/2, 1:1500, Cell Signaling Technology; and mouse anti-β-actin, 1:2000, Tianjin Sungene Biotech Co., Ltd) and with appropriate secondary antibodies for 1 h at room temperature (goat anti-rabbit IgG and goat anti-mouse IgG, 1:2000, Cell Signaling Technology). The signals of protein bands were detected by enhanced chemiluminescence reagent (Applygen). Band intensity was normalized with β-actin as the endogenous control. And for the detection of phosphorylation of JNK1/2 and Erk1/2, the membrane was regenerated by regenerative liquid (Applygen) and went through another time of defatted milk blockage, primary antibody incubation, secondary antibody incubation and protein band detection. Results of JNK1/2 and Erk1/2 were shown by a comparison of phosphorated to total after normalized by β-actin.

### Statistical analysis

The quantitative results were presented as mean ± standard error in the bar graph. And the statistical method used was analysis of variance of factorial design and one-way analysis of variance (ONE-WAY ANOVA) using SPSS 21.0. The significant difference was shown by * and #, which represented the values of *P* < 0.05. N.S stands for no statistical difference.

## Results and discussion

### Characterization of PAMAM-GSH

GSH depletion in the dopamine neurons is a common feature of patients with PD. For GSH is synthesized inside neurons and astrocytes to perform its ability as an antioxidant and neuromodulator, we took advantage of PAMAM dendrimer’s transmembrane ability and high load performance to synthesize PAMAM-GSH conjugates with GSH.

**Figure 1.**
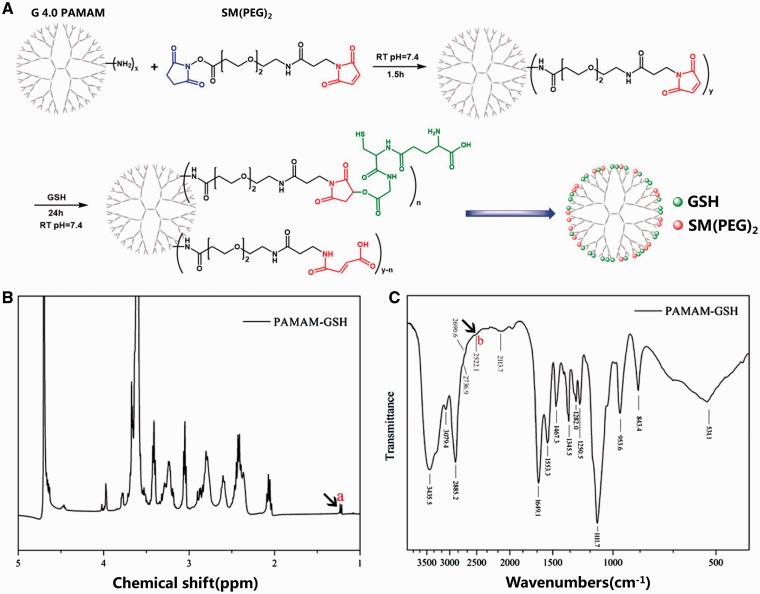
Characterization of PAMAM-GSH. (**A**) the reaction scheme of PAMAM dendrimer and GSH for PAMAM-GSH synthesis. ^1^H NMR spectrum (**B**) and FTIR spectra (**C**) of PAMAM-GSH.

To synthesize generation 4.0 PAMAM-GSH, we used N-hydroxysuccinimide-PEG_2_-maleimide(SM(PEG)_2_) as crosslinker to get PAMAM-SM(PEG)_2_ to facilitate the reaction of GSH and PAMAM dendrimer (Fig. 1A). After further purification by dialysis and lyophilization, the successful combination of GSH onto PAMAM-SM(PEG)_2_ was confirmed by ^1^H NMR and FTIR analysis. As indicated from other researches, the characteristic peak of H-S of GSH is at 1.2 ppm for ^1^H NMR and 2522 cm^−1^ for FTIR [36, 37]. As shown in Fig. 1B and C, there were characteristic peaks of GSH in the analysis of PAMAM-GSH at 1.2 ppm in ^1^H NMR and 2522.1 cm^−1^ in FTIR analysis, which indicated the successful reaction of GSH onto PAMAM-SM(PEG)_2_. Besides, the characteristic carbonyl bond of GSH at 1720 cm^−1^ was disappeared in FTIR analysis of PAMAM-GSH, which suggested the reaction of GSH to PAMAM-SM(PEG)_2_ was between carboxylic groups of GSH and maleimide groups of SM(PEG)_2_ (Fig. 1C).

### Cytotoxicity of PAMAM-GSH

**Figure 2.**
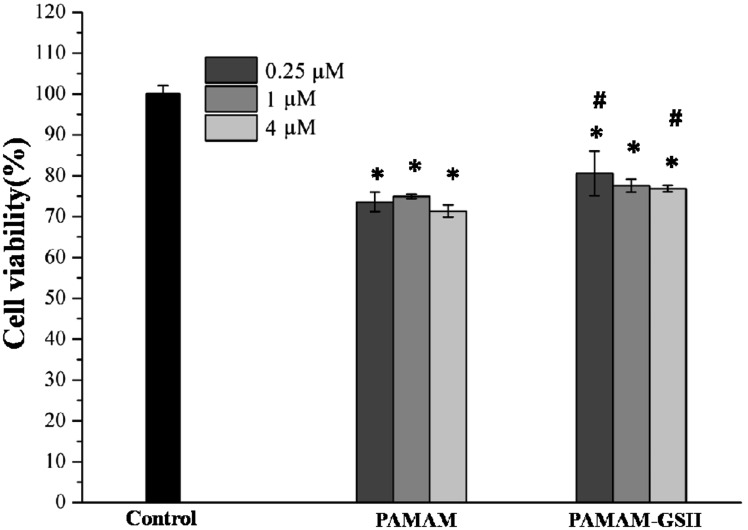
Cytotoxicity of PAMAM dendrimer and PAMAM-GSH to PC12 cells. PC12 cells were pre-incubated with PAMAM dendrimer and PAMAM-GSH, respectively for 24 h before MTT assessment. * indicates significant difference compared to control (*P* < 0.05). # indicates significant difference between PAMAM dendrimer group and PAMAM-GSH group of the same concentration (*P* < 0.05).

As illustrated from other researches that PAMAM dendrimer can induce cytotoxicity depending on the concentrations, the generations, the electric charges as well as the functional groups of PAMAM dendrimer [38–41]. MTT assessment is often used for the cytotoxicity assessment to indicate the cellular metabolic ability. So MTT assessment was utilized to analyze the cytotoxicity of PAMAM-GSH. As shown in Fig. 2, PAMAM dendrimer and PAMAM-GSH of different concentrations had a lower cellular metabolic ability than control (*P* < 0.05), which indicates that both PAMAM dendrimer and PAMAM-GSH can interfere with the metabolic activity of PC12 cells. Moreover, the same concentration of 0.25 and 4 μM PAMAM-GSH showed higher metabolic activity than PAMAM dendrimer group (*P* < 0.05), which may due to the protective effect of GSH. Therefore, further experiment is required for identifying the protective role of PAMAM-GSH dendrimer.

### Analysis of GSH delivery

Here, FCA, DTNB intracellular GSH detection, (TEM) and confocal microscopy were used to analyze the efficiency of PAMAM-GSH delivery ability.

**Figure 3.**
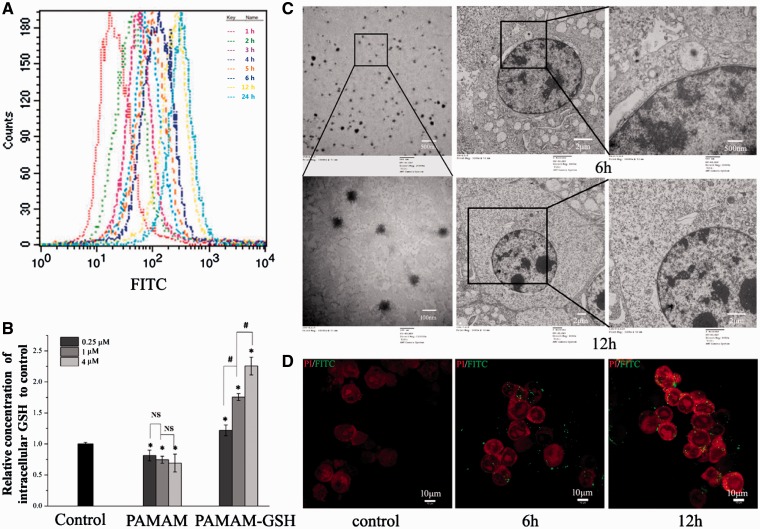
Characterization of the intracellular GSH delivery effect of PAMAM-GSH to PC12 cells. (**A**) FCA of 1 μM PAMAM-GSH to PC12 cells for 1–24 h as indicated in methods. (**B**) DTNB method for the analysis of intracellular concentration of GSH in PC12 cells after incubation with different concentrations of PAMAM-GSH and PAMAM dendrimer for 24 h. (**C**) (TEM) analysis of PAMAM-GSH in PBS (left), and 1 μM PAMAM-GSH to PC12 cells for 6 and 12 h. (**D**) confocal microscopy of 1 μM FITC-labeled PAMAM-GSH to PC12 cells for 0, 6 and 12 h. * indicates significant difference compared with control (*P* < 0.05). # indicates significant difference between groups of PAMAM-GSH (*P* < 0.05).

For FCA, we adopted 1 μM FITC-Labeled PAMAM-GSH to study its delivery ability of GSH onto or into PC12 cells. As showed in Fig. 3A, the average fluorescence intensity became stronger as the incubation time goes from 1 to 24 h, which indicated a time dependent manner of PAMAM-GSH. And then we performed intracellular GSH analysis of different concentrations of PAMAM-GSH and PAMAM using DTNB method [42]. As shown in Fig. 3B, compared with control, the relative concentrations of intracellular GSH delivered into PC12 cells with different concentrations of PAMAM-GSH were higher (*P* < 0.05). And the higher concentration of PAMAM-GSH is, the higher concentration of intracellular GSH were shown (*P* < 0.05). To further examine how PAMAM-GSH delivered GSH into PC12 cells, we chose PAMAM-GSH of 1 μM to perform TEM analysis. As illustrated from other researchers, PAMAM dendrimer goes into cells by endocytosis and localizes in cytoplasm [43]. The results in Fig. 3C indicated that PAMAM-GSH was internalization by PC12 cells and finally localized in the cytoplasm, which is consistent with previous researches. And we also conducted a confocal microscopy analysis of FITC-labeled PAMAM-GSH of 1 μM to PC12 cells with incubation time from 0 to 12 h, which showed that the FITC fluorescence located around the nuclear of PC12 cells and became stronger when the incubation time went from 0 to 12 h (Fig. 3D). According to the results above, we concluded that PAMAM-GSH could efficiently deliver GSH into PC12 cells and PAMAM-GSH of higher concentration showed much better delivery ability. Besides, PAMAM-GSH could be internalized by PC12 cells and localized in the cytoplasm around the nuclear.

### MPP-induced PC12 cellular model of PD

**Figure 4.**
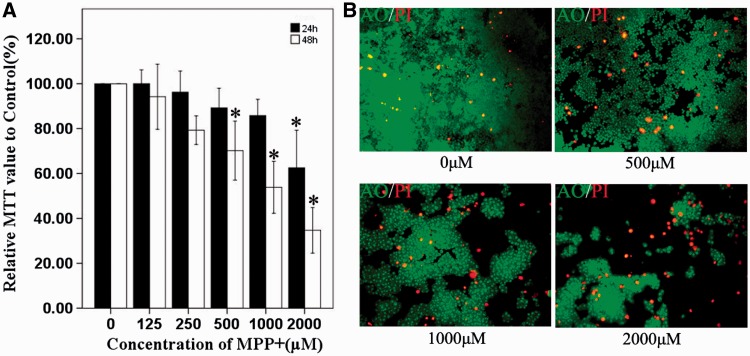
MPP-Induced PC12 cells as an oxidative stress model for PD. (**A**) MTT assessment of MPP-induced PC12 cells. (**B**) AO/PI staining of PC12 cells after 500–2000 μM MPP incubation for 48 h, while the control was incubated with normal culture medium (top, left). * indicates significant difference to control (*P* < 0.05).

There are several cellular models of PD which include SH-SY5Y [44], PC12 cells [45], embryonic carcinoma cell line [46] and stem cells [47–49]. In order to find out if PAMAM-GSH shows protective ability from oxidative stress, we used MPP-induced PC12 cells as an oxidative stress model for PD. Although the cellular model of MPP-induced oxidative stress is often used, the concentrations and incubation time of MPP used in different models varied [50, 51]. As shown in Fig. 4A, we used different concentrations of MPP which ranges from 125 to 2000 μM to treat PC12 cells for 24 and 48 h to explore the best oxidative effect on PC12 cells. Compared with control group (0 μM), PC12 cells in 125 and 250 μM MPP groups showed no difference all the time. However, PC12 cells in 2000 μM group showed lower MTT value than the control group when incubated for 24 and 48 h (*P* < 0.05) and PC12 cells of 500 μM as well as the 1000 μM group showed lower MTT value than the control group when incubated for 48 h (*P* < 0.05). It was concluded from the MTT assessment that 500 μM MPP showed proper metabolic viability of PC12 cells, which was about 70% of the control. To further analyze the influence of 500–2000 μM MPP on the cell density and percentage of dead PC12 cells, we performed AO/PI staining experiment. As shown in Fig. 4B, 500 μM, 1000 μM and 2000 μM MPP displayed much higher dead cell percentage and much lower cellular densities than the control group. To establish a proper MPP-induced oxidative stress model of PC12 cells, the group that best mimics the oxidative stress injury should be chosen while does not influence the coming process of living in PC12 cells. Analyzing the results showed that PC12 cells treated with 500 μM MPP for 48 h have the best suitable dead cell percentage and metabolic viability. And then we chose to use 500 μM MPP to treat PC12 cells for 48 h to prepare the subsequent oxidative stress cellular model of PD.

### Antioxidative property of PAMAM-GSH

**Figure 5.**
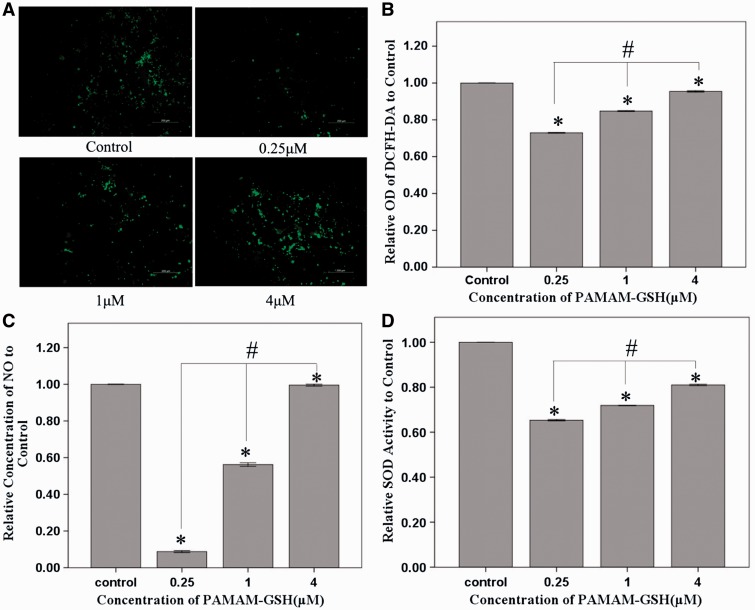
Anti-Oxidative ability of PAMAM-GSH to MPP-induced PC12 cells. (**A**) DCFH-DA staining of intracellular ROS produced inside PC12 cells. (**B**) Absorption value of DCFH-DA staining for intracellular ROS assessment. (**C**) Relative concentration of NO released from PC12 cells with different concentrations of PAMAM-GSH treatment. (**D**) Relative intracellular SOD activity of PC12 cells with different concentrations of PAMAM-GSH treatment. * indicates significant difference compared with control (*P* < 0.05). # indicates significant difference between groups of PAMAM-GSH (*P* < 0.05).

MPP-induced oxidative injury is performed by the inhibition of complex I function of mitochondria [17]. ROS levels increased in the induction of MPP. Besides, ROS plays an important role in PD [52]. To analyze the protective effect of PAMAM-GSH, PC12 cells were incubated with different concentrations of PAMAM-GSH for 24 h before adding 500 μM MPP for another 48 h. The antioxidative ability of PAMAM-GSH was performed by the detection of intracellular ROS, the release of intracellular NO and the activity of intracellular SOD. PAMAM-GSH showed significant inhibition effect of intracellular ROS and PAMAM-GSH of 0.25 μM showed the best inhibition ability of intracellular ROS (Fig. 5A and B). Similarly, PAMAM-GSH inhibited the release of intracellular NO and reduced the levels of intracellular SOD and PAMAM-GSH of 0.25 μM showed the most effective protection (Fig. 5C and D). Therefore, PAMAM-GSH of 0.25 μM showed the best antioxidative ability.

### Antiapoptotic ability of PAMAM-GSH

**Figure 6.**
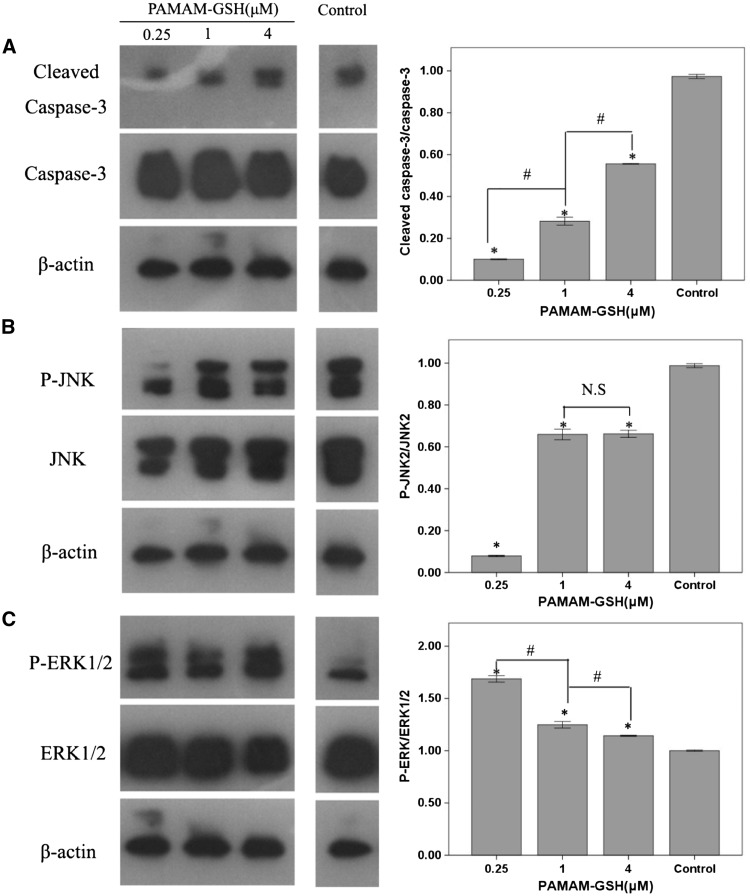
Anti-Apoptotic ability of PAMAM-GSH to MPP-induced PC12 cells. (**A**) western blot analysis of caspase-3 and cleaved caspase-3 expression of different concentrations of PAMAM-GSH, (**B**) western blot analysis of JNK1/2 protein expression, (**C**) western blot analysis of Erk1/2 protein expression. * indicates significant difference from control (*P* < 0.05), # indicates significant difference between groups of PAMAM-GSH (*P* < 0.05), N.S stands for no significant difference between groups.

Apoptosis involved in PD has been verified by post-mortem and other techniques [53, 54]. In order to further prove the protective effect of PAMAM-GSH, western blot analysis of caspase-3 and cleaved caspase-3 was used for antiapoptotic assessment. As showed in Fig. 6A, compared with control, PAMAM-GSH of different groups showed lower level expression of cleaved caspase-3 (*P* < 0.05). Moreover, there is a significant difference between groups for cleaved caspase-3 expression in which PAMAM-GSH of 0.25 μM showed the least level of cleaved caspase-3 expression (*P* < 0.05). The expression of cleaved caspase-3 is employed for cellular apoptosis assessment and a higher level expression of cleaved caspase-3 indicates the higher level of apoptosis. Therefore, the lowest level of apoptosis indicated the best protective effect of PAMAM-GSH from cellular apoptosis. It was indicated from other researches that JNK2 and Erk1/2 pathways have involved in the apoptosis of PD [55, 56]. To further analyze which pathway has been involved in the protective role of PAMAM-GSH against apoptosis, western blot assessment of Erk1/2, JNK1/2 was performed. It was demonstrated that the expression of phospho-JNK2 was lower than control while phospho-Erk1/2 was higher than control (Fig. 6B and C). From the above results, we could find that the lower concentration of PAMAM-GSH possessed the stronger antiapoptotic ability and PAMAM-GSH of 0.25 μM showed the best antiapoptotic ability. Also, we found that JNK2 and Erk1/2 signal pathway has been involved in the antiapoptotic effect of PAMAM-GSH on MPP-induced cellular apoptosis.

## Conclusion

From the above data, we could find that GSH has been successfully conjugated onto PAMAM dendrimer which was confirmed by the characteristic peaks of GSH shown in ^1^H NMR and FTIR analysis. PAMAM-GSH showed a lower cytotoxic effect on PC12 cells than the pure PAMAM dendrimer indicates the protective role of GSH conjugated onto PAMAM dendrimer. PAMAM-GSH can be internalized into the cytoplasm by PC12 cells and showed its delivery effect of GSH by a concentration dependent manner in which PAMAM-GSH of higher concentration exhibited a better delivery ability. At the same time, PAMAM-GSH decreased the intracellular level of ROS induced by MPP and inhibited the activation of caspase-3. As indicated from other researches, the cationic amino groups contribute to the membrane binding ability as well as the cytotoxic effect of G4.0 PAMAM dendrimer [45]. So the cytotoxic effect, antioxidative and antiapoptotic ability of PAMAM-GSH may combine the roles of GSH and amine groups. Though PAMAM-GSH of higher concentration exhibited better delivery of GSH into PC12 cells, it also raised the cytotoxicity and reduced its antioxidative and antiapoptotic ability because of the improper percentages of amino groups and GSH. As a result, PAMAM-GSH of lower concentration showed much better antioxidative and antiapoptosis ability. Due to its significant antioxidative and antiapoptotic ability, PAMAM-GSH can be used in the therapy of PD. In the further research, PAMAM-GSH dendrimer with higher substituent degree of GSH will be prepared for more efficient antioxidative and antiapoptotic effect.

## Funding

This study was supported by Program of International Scientific and Technological Cooperation and Exchanges of China (No. 2013DFG30680), National Key Research and Development Program (2016YFC1101303), Beijing Natural Science Foundation (No. 7162142) and National Natural Science Foundation of China (No. 81272912).

*Conflict of interest statement.* None declared.
